# Veratrum Viride in Puerperal Eclampsia

**Published:** 1886-12

**Authors:** U. G. M. Walker

**Affiliations:** Tyler, Texas


### VERATRUM IN PUERPERAL ECLAMPSIA.

*By U. G. M. Walker, M. D., Tyler, Texas.*

(For Daniel's Texas Medical Journal.)

GESTATING, as well as parturient women, are unfortunately predisposed to many ills, and among them none is more appalling or attended with more unfortunate termination than puerperal convulsions. Indeed, this dread manifestation of perturbed nervous action, is one of the most distressing that the accoucheur has to encounter.

Though recognized and described from the earliest chronicles of obstetric records, the *treatment* has by no means been uniform, excepting the time-honored advice to *bleed.* Not that we would criticise or ignore this advise, for certainly it is an agent of great power, and much good in many cases, and under certain conditions of system it is *the* agent to accomplish all the good that a remedy can do. There are other agents too, to supplement its action, and in some instances to take its place, that successfully carry a patient through the perils of this dread ordeal—chloroform, hydrate of chloral, bromide pososssium, gelsemium, lobelia *et id omne genus*, have been resorted to, and favorable reports of successful results attributed to their use. But in this brief article I wish particularly to call the attention of practitioners to the beneficial action, and result from the use of that great *nervous* and *circulatory sedative,* Veratrum-Viride.

Not that use of this agent is new, or original with me, in the treatment of this malady, but as its use was attended with favorable results, and as all testimony in the use of a remedy is valuable in establishing its value, I therefore report the following case:

On the night of December —, I was called to see Mrs. P——, a primipara, aged about 20, who, after retiring, was seized with a convulsion. When I arrived she was quiet, and counsciousness had returned. She was anasarcous, with considerable peritoneal effusion. The pleural cavities and pericardal sac, I found, upon physical examination, to be free from any dropsical accumulation. Her time of confinement was near, but upon vaginal examination there was evidence of actual labor. I found, upon inquiring into the history of the case, that for some two or three months she had occasionally had chills, and for some six weeks had gradually become dropsical; the urine, upon examination, proved to be *highly* albuminous. I gave an emetic of Ipecac, and she vomited freely an undigested mass; this was followed by an hypodermic of morphine, ¼ grain. She was quieted, slept well, and for three days continued up. On the morning of the — I was called, again and found that she had two convulsions. Examination now showed that labor had commenced. Gave chloroform by inhalation which, though mitigating the convulsions, did not entirely arrest them. They continued to recur at intervals of an hour, subsequently at an interval of half an hour, and the paroxysms shortened to from 15 to 20 minutes. At this stage my professional friend, Dr. D. H. Conally, was called. and upon consultation, as the *first* stage of labor was not completed, the os uteri undilated, and the head *above* the superior strait, the pains feeble, we determined upon the *combined* action of Ergot and Veratrum, giving them in combination hypodermically, fifteen drops of the former, to ten of the latter; this repeated at an interval of an honr, quieted the convulsions and increased the uterine action; the convulsions ceased after the third dose, labor progressed and by the *natural* forces of labor a dead fœtus was delivered. Though unconscious when the delivery took place, and remaining so for some 24 hours, the mother has daily improved and is now fully convalescent, the dropsical condition having disappeared. In this case some 25 or 30 convulsions had occurred, and were recurring, the interval shortening between the paroxysms, up to the time we exhibited the Veratrum and Ergot. I am perfectly satisfied that the cessation of the convulsive paroxysms was due to the sedative effects of the Veratrum.

The Ergot undoubtedly stimulated uterine action and hastened the termination of labor. It may be asked why was the obstetric forceps not used. We had them at hand, but fortunately when the os was sufficiently dilated, or even dilatable, to *prudently* apply them, active uterine action occurred, the membranes ruptured, and two or three decided pains terminated the labor. While we are a decided advocate for the proper use of this invaluable obstetric aid, and particularly in convulsions, we will never be so bold as to introduce into an undilated os, or even attempt to grasp a head above the superior strait. At the stage when we intended to apply them, uterine action was, fortunately, so vigorous as not to necessitate their use.

